# Comparison of Photocatalytic and Photosensitized Oxidation of Paraben Aqueous Solutions Under Sunlight

**DOI:** 10.1007/s11270-018-3991-y

**Published:** 2018-10-26

**Authors:** M. Foszpańczyk, K. Bednarczyk, E. Drozdek, R. C. Martins, S. Ledakowicz, M. Gmurek

**Affiliations:** 1 Department of Bioprocess Engineering, Faculty of Process and Environmental Engineering, Lodz University of Technology, Wolczanska 213, 90-924 Lodz, Poland; 2 Department of Safety Engineering, Faculty of Process and Environmental Engineering, Lodz University of Technology, Wolczanska 213, 90-924 Lodz, Poland; 3 CIEPQPF – Chemical Engineering Processes and Forest Products Research Center, Department of Chemical Engineering, Faculty of Sciences and Technology, University of Coimbra, Rua Sílvio Lima, 3030-790 Coimbra, Portugal

**Keywords:** Photocatalysis, Photosensitized oxidation, Paraben photodegradation, Solar water disinfection

## Abstract

It is well-established that aquatic wildlife is exposed to natural and synthetic endocrine disrupting compounds which are able to interfere with the hormonal system. Although advanced oxidation processes (AOPs) have shown to be effective, their application is limited by a relatively high operational cost. In order to reduce the cost of energy consumed in the AOPs, widely available solar energy instead of UV light may be applied either as photocatalytic oxidation or as photosensitized oxidation. The main goal of the present study was to investigate the sunlight photodegradation of paraben mixture. Two processes, namely the photocatalytic oxidation with modified TiO_2_ nanoparticles and photosensitized oxidation with photosensitive chitosan beads, were applied. The oxidants were identified as singlet oxygen and hydroxyl radicals for photosensitized and photocatalytic oxidation, respectively. The toxicity, as well as ability to water disinfection of both processes under natural sunlight, has been investigated. Application of sunlight for the processes led to degradation of parabens. The efficiency of both processes was comparable. Despite the fact that singlet oxygen is weaker oxidant than hydroxyl radicals, the photosensitized oxidation seems to be more promising for wastewater purification, due to the possibility of chitosan bead reuse and more effective water disinfection.

**Electronic supplementary material:**

The online version of this article (10.1007/s11270-018-3991-y) contains supplementary material, which is available to authorized users.

**Graphical Abstract:**
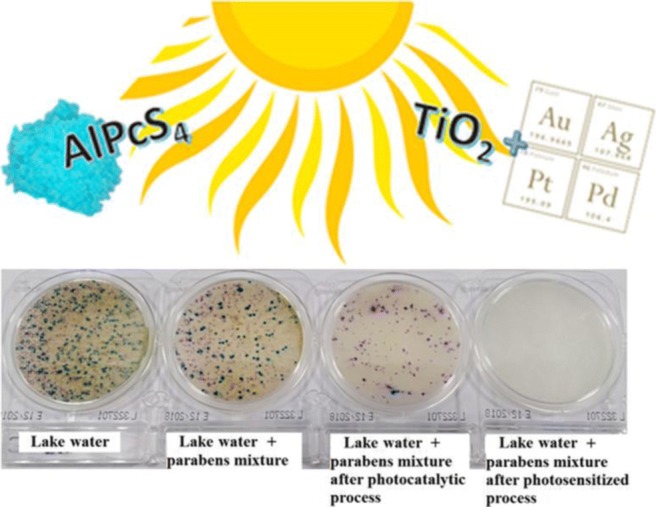
ᅟ

## Introduction

In recent years, environmentally friendly methods for the removal of organic and inorganic impurities from water have been intensively investigated. For the last decades, the scientific attention has been paid to highly effective advanced oxidation processes (AOPs) which include photochemical processes involving UV or VIS light (Gmurek et al. 2017). However, the visible light application is only possible in photocatalytic or photosensitized oxidation (Gmurek et al. 2017). Radiation may cause transfer of energy—photosensitization (photoexcitation of the photosensitizer with charge transfer towards the substrate in the basic state) or photocatalysis reaction (excitation with the transfer of charge to the catalyst) (Petala et al. 2015; Papadopoulos et al. 2016).

Modified photocatalysts based on TiO_2_, which have higher activity under visible light, can be prepared by TiO_2_ doping with metals or sensitizer dyes (absorbing visible light). The redox potential of the substance in the presence of a catalyst oxidized must be located above the valence band of the semiconductor (Gmurek et al. 2017). The absorption of the radiation of suitable energy (greater than or equal to the energy band gap) excites an electron from the valence band to the conduction band. Photoexcitation generates highly oxidizing holes and reducing electrons. Reaction on the surface of the photocatalyst is a redox reaction (Giraldo-Aguirre et al. 2015). However, the oxidation of the substrate occurs as a result of hydroxyl radical (HO·) attack, generated at the TiO_2_ surface.

The photosensitized oxidation does not require modification of photosensitizer to be active to generate singlet oxygen (^1^O_2_), which is the main oxidant under visible light. The photosensitizer can be excited only by this kind of radiation, what is unquestionably an advantage of this process (Gmurek et al. 2017). Anyhow, the oxidative ability of ^1^O_2_ is much lower than the one of HO·. In both cases, the photodegradation process depends on the intensity of light, adsorption of pollutants on catalysts/carrier surface, pH, amount of anions and cations in reaction solution, the reaction temperature, and the surface area of the photocatalyst (Martins and Quinta-Ferreira 2009; Chuang and Luo 2015; Gmurek et al. 2015, Gmurek et al. 2017).

The solar water disinfection is well known since 1980; however, over recent years, it has been developed considerably (Berney et al. 2006; Boyle et al. 2008; Ghanizadeh et al. 2015; Castro-Alférez et al. 2016). Therefore, when the application of natural solar light is considered, the possibility of solar water disinfection occurring simultaneously with photodegradation of organic compounds should be investigated.

This article is focused on the comparison of photocatalytic and photosensitized oxidation of hazardous aquatic pollutants—parabens. Parabens are a group of organic compounds (esters of para-hydroxybenzoic acid) used in cosmetics, pharmaceutical products, and food (Golden et al. 2005). They display antibacterial (especially in the case of Gram-positive bacteria) and antifungal properties. These compounds are stable and resistant to decomposition under the influence of water and acid solutions. In an acidic environment, they exhibit activity against yeasts and molds (Golden et al. 2005; Charnock and Finsrud 2007), but they are not enough effective against bacteria (Lin et al. 2009, 2011; Fang et al. 2013). The mechanism of action of parabens is multi-way. Parabens are classified as endocrine disrupting compounds that actively influence the hormonal system of organisms. Additionally, their association with various kinds of allergies and the malfunctioning of reproductive organs has been found (Raza et al. 2018). It should be noted, however, that the strongest effect occurs at the cells of microorganisms by disrupting the transmembrane transport processes and through the lipid membrane building system disorders (Golden et al. 2005; Charnock and Finsrud 2007; Uysal and Güray 2008). Table 1 shows the summary patterns of popular parabens.

**Table 1:**
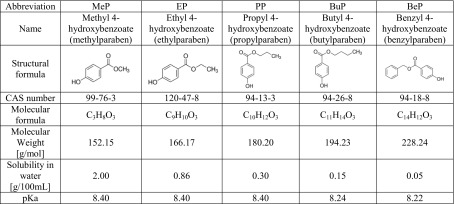
The characteristics of parabens (Gmurek et al. 2015; Yalkowsky et al. 2010)

Parabens are solid, finely structured, colorless, and odorless. With the increase in the chain length of the alkyl group, the antiseptic activity of the parabens increases and their solubility in water decreases. Parabens show no preservative properties in the oil phase. Individual parabens exhibit different preservative properties. Moreover, they show the best preservative action when applied in mixtures. According to the EU directive 1223/2009, the concentration of parabens in the cosmetic products must not exceed 0.4 and 0.8% of single ester and mixtures of esters, respectively. Since 2015, isobutyl 4-hydroxybenzoate, isopropyl 4-hydroxybenzoate, phenyl 4-hydroxybenzoate, benzyl 4-hydroxybenzoate, and pentyl 4-hydroxybenzoate are prohibited ingredients (Official Journal of the European Union 2014). Moreover, propyl- and butyl 4-hydroxybenzoate permitted loads were reduced to 0.14%. Besides, such products were prohibited for use in products for children under 3 years (Official Journal of the European Union 2014).

The main goal of the present study was to compare two solar-driven photochemical processes: photocatalytic and photosensitized oxidation for paraben mixture degradation. Both processes were carried out in a heterogeneous system. Photosensitized oxidation was performed in the presence of photoactive chitosan, while photocatalytic oxidation was examined in an aqueous suspension of the catalyst. The noble metals were used for modification of TiO_2_, while photosensitive chitosan beads were applied as an insoluble carrier for photosensitizer immobilization. *Vibrio fischeri* luminescence inhibition was used to determine the changes of toxicity activity in the mixture during natural sunlight irradiation. The sensitivity of coliform and pathogenic enteric strains *Salmonella* to photosensitized and photocatalytic oxidation under natural sunlight was also investigated.

To the best of our knowledge, this is the first time that comparison of noble-metal doped TiO_2_ catalysts and photosensitive chitosan beans is applied in photocatalytic as well as photosensitized oxidation under natural sunlight condition for paraben degradation. Moreover, the analysis of the degradation of a mixture rather than individual compounds is a novel feature of the work. Furthermore, the evaluation of photodegradation was performed on several levels: (i) monitoring of paraben decay over time, (ii) comparison of toxicity of experimental mixture before and after irradiation, and (iii) disinfection potential of both methods.

This approach provides more realistic data regarding the behavior of treatment processes when applied for the degradation of real wastewaters containing these dangerous compounds and other organic compounds. It is well known that the matrix in which the photodegradation processes are conducted affects the efficiency of the process (Gmurek et al. 2015; Gomes et al. 2019). The main novelty of this work is the use of natural solar radiation for activating the photocatalyst as well as photosensitizer immobilized into chitosan in a reaction system with potential environmental engineering applications. Comparison of two photoprocesses from ecotoxicology and microbiological water quality point of view, using environmental samples (lake water), adds novelty to this study. Since the treatment processes proposed are not able to totally mineralize organic matter, it is essential to understand the potential effect of the resulting treated effluents on the ecosystems and to predict the possible positive or negative effects.

## Material and Methods

### Materials

Methyl 4-hydroxybenzoate 99% (MeP), propyl 4-hydroxybenzoate 99% (PP), and benzyl 4-hydroxybenzoate 99% (BeP) were purchased from Sigma-Aldrich, ethyl 4-hydroxybenzoate 99% (EP) from Aldrich, and butyl 4-hydroxybenzoate 99% (BuP) from Fluka. These compounds were used as model aqueous pollutants. All reagents used in this work were of analytical grade and were used without any further purification. All solutions were prepared using water from a Millipore system (Direct-Q® Water Purification System- Merck Millipore).

Titanium(IV) isopropoxide (TIP) (97%) was purchased from Aldrich and used as a titanium source for the preparation of TiO_2_ nanoparticles. A commercial form of TiO_2_ (P25, crystalline composition: 80% anatase, 20% rutile, surface area 50 g/m^2^) was obtained from Evonik, Germany. PdCl_2_ (5 wt% solution in 10 wt% HCl), NH_2_NH_2_·H_2_O (reagent grade 50–60%), H_2_PtCl_6_ (99%), and AgNO_3_ (99%) from Sigma-Aldrich were used as metal source in the preparation procedure. Ethanol (99.8%) was purchased from POLCHEM (Poland).

A photosensitizer (aluminum phthalocyanine chloride tetrasulfonic acid, AlPcS_4_) from Frontier Scientific was immobilized in chitosan beads. Chitosan from crab (degree deacetylation ≥ 75%) used for carrier preparation was purchased from Sigma. Na_2_HPO_4_, NaOH, and all of p.a. quality (POCh, Poland) were used to prepare buffer solutions at pH 9.

### Methods

#### Preparation of Catalysts and Photosensitive Chitosan

##### Chitosan Preparation

The photoactive chitosan carrier was prepared in the form of 3–4 mm beads. Hydrogel beads were produced by the phase inversion method from an aqueous solution of chitosan in acetic acid. The chitosan solution was dropped from 0.8 mm needle into NaOH, where coagulation was carried out. Photosensitizer immobilization into the chitosan carrier was carried out by adsorption of AlPcS_4_ from the aqueous solution.

##### Catalysts Preparation

Five catalysts (pure TiO_2_, TiO_2_-Pt, TiO_2_-Pd, TiO_2_-Ag, and TiO_2_-Au) were applied. All doped photocatalysts were obtained by the UV reduction of Pt^4+^, Pd^2+^, Ag^+^, and Au^3+^ ions in the TiO_2_ suspension. An aqueous solution of isopropanol containing H_2_PtCl_6_ (0.5 wt%) or PdCl_2_ (0.5 wt%) was degassed with nitrogen and irradiated by UV-Vis light (1000 W Xe lamp) for 6 h according to Klein et al. (2016). Aqueous solutions of ethanol containing AgNO_3_ (0.5 wt%) and AuCl_4_K (0.5 wt%) were degassed with nitrogen and irradiated by UV-Vis light (1000 W Xe lamp) for 100 min. The modified TiO_2_ photocatalysts were separated by centrifugation and dried at 65–120 °C for 12 h. TiO_2_-Pt, TiO_2_-Pd, and TiO_2_-Ag were prepared via the photodeposition while TiO_2_-Au via a sol-gel method with thermal treatment process—calcination at 400 °C according to the procedures presented by Stelmachowski et al. (2014a, b) and Gomes et al. (2017, b). The average diameter of photocatalyst particles was in the range 6–8 nm depending on the kind of noble metal-doped.

#### Photodegradation—an Experimental Procedure

Both photodegradation processes were carried out mostly in demineralized water from the Millipore Milli-Q Plus System (18.2 MΩ) (pH 6.3–6.5). Moreover, some experiments were performed in tap water (pH ~ 5) and natural water (pH 5.5) from a lake in Pabianice (Poland, 51°38′50.3″ N 19°22′19.7″ E) in which parabens were spiked. Furthermore, photosensitized oxidation was examined also in a buffered solution at pH 9.

Both photodegradation processes were conducted under simulated (lamp) as well as natural sunlight radiation.

All of the experiments were repeated at least twice under the same reaction conditions.

##### Photocatalytic Oxidation

Photocatalytic oxidation was performed under a xenon lamp (Xe lamp) and natural sunlight radiation. Several kinds of reactors were used. Firstly, photodegradation reactions were performed in a glass semi-batch reactor (1200 mL) with quartz immersion cooling well where the source of light was positioned. The working volume of the reaction solution was equal to 800 mL. The internal Xe lamp (100 W, 260 W/m^2^, 6.49 × 10^−5^Einstein/(Ls)) was used to simulate sunlight. The reaction mixture was purged with air. Then, the collected samples were filtrated via 0.45-μm nylon filters (ChemLand). The prepared and filtered samples were analyzed on the HPLC. The initial temperature of the reaction mixture was 25 °C and during the experiments increased to 30 °C. Additionally, tests were carried out also on the smaller reactor volume (0.25 L) with two Xe lamps (each 75 W, 3.20 × 10^−5^ Einstein/(m^2^s)), which were externally located. The schemes of the quartz reactors are shown in Fig. S1. The concentration of each paraben was 10 mg/L. The amount of catalysts was equal to 70 mg/L.

Based on our previous research (Gomes et al. 2017, b, c, 2019), it was concluded that among the five parabens, MeP and EP are the most resistant to degradation. Moreover, those two compounds are the mostly used parabens. Thus, for the preliminary study (examination of metal doping, type of reactor, the position of the light source), only two parabens (MeP and EP) were degraded. When the effect of different water matrix under natural sunlight was investigated, all five parabens were tested.

##### Photosensitized Experiments

Photoreactor with external sources of light was employed. Simulated light by high-pressure sodium lamp (Lumatek, 400 W, 222.6 W/m^2^, 1.44 × 10^−3^ Einstein/(m^2^s)) and natural sunlight were used to excite the photosensitizer. The experiments in a semi-batch mode were carried out in a 0.5-L glass reactor with a cooling jacket, equipped with a porous plate to disperse gas into the reaction solution (Fig. S2). The reaction mixture was aerated and agitated by gas bubbling, and the temperature was maintained at 20 °C. When photoactive chitosan carrier was put into the reactor, the reaction solution was added. The concentration of each of five parabens in a solution was 10 mg/L. Fifty-five grams of photoactive chitosan was suspended in 0.5 L of the reaction solution.

##### Sunlight Experiments

Sunlight irradiation experiments were performed on sunny days (June/July 2016 and repeated in June/July 2017, Poland, 51°45′14.2″ N 19°27′12.9″ E). The presented results are average values of concentration obtained from both series of experiments (error from mean value was from 2 to 6%). The light spectra were acquired with an Oceans Optics USB 4000 fiber optic spectrometer with an approximate resolution of 0.4 nm. The average irradiance for photosensitized experiments with MQ was around 280 W/m^2^ (1.37 × 10^−3^Einstein/(m^2^s)), with lake water 340 W/m^2^ (2.04 × 10^−3^ Einstein/(m^2^s)), with tap water 230 W/m^2^ (1.02 × 10^−3^ Einstein/(m^2^s)). The photocatalytic oxidation process under sunlight was carried out on a slightly cloudy day; therefore, the irradiance values were from 74 W/m^2^ (3.45 × 10^−4^/(m^2^s)) to 489 W/m^2^ (2.69 × 10^−3^/(m^2^s)).

The reactor used during sunlight experiments is shown in Fig. S2.

#### Analytical Methods

The reaction efficiency was monitored by determination of paraben concentration using Agilent 1220 Infinity LC HPLC apparatus. Separation of the analytes was done on a Poroshell 120 C18 column (2.7 μm). The method involved gradient elution with methanol with 0.1% formic acid (A) and water with 0.1% formic acid (B), with the flow rate of 0.7 ml/min. The gradient method is presented in the Table S1. The injection volume for all samples was 10 μl, and all the compounds were monitored at 257 nm with a DAD detector. The compounds in the mixture were analyzed in one injection and characterized by their respective retention times.

The anion concentrations in water matrixes were determined by an ion chromatograph (ICS-1100, DIONEX, AR, USA) on an IonPac AS18 column.

#### Ecotoxicity Assessment

The acute ecotoxicity bioassay was conducted using a Microtox Model 500 analyzer (Modern Water, New Castle, DE, USA) with the marine bacterium *Vibrio fischeri* as a bioluminescent indicator. Microtox® 81.9% Basic Test protocols available with the MicrotoxOmni™ analyzer software were used for the ecotoxicity assessment of samples.

#### Microbiological Water Quality Analysis

The microbiological analysis of the samples of lake water, as well as paraben solution, was performed. In a presented study, specific compact plates were used to detect Coliform (*Escherichia coli* and *Enterobacter aerogenes*) (Compact Dry “Nissui” EC, Nissui Pharmaceutical Co., Tokyo, Japan) as well as *Salmonella* (Compact Dry “Nissui” SL, Nissui Pharmaceutical Co., Tokyo, Japan). For the Compact Dry SL and Compact Dry EC tests, the incubation time was 24 h, while the temperatures were 42 ± 1 °C and 35 ± 2 °C, respectively.

## Results and Discussion

### Photosensitized Oxidation

**Fig. 1:**
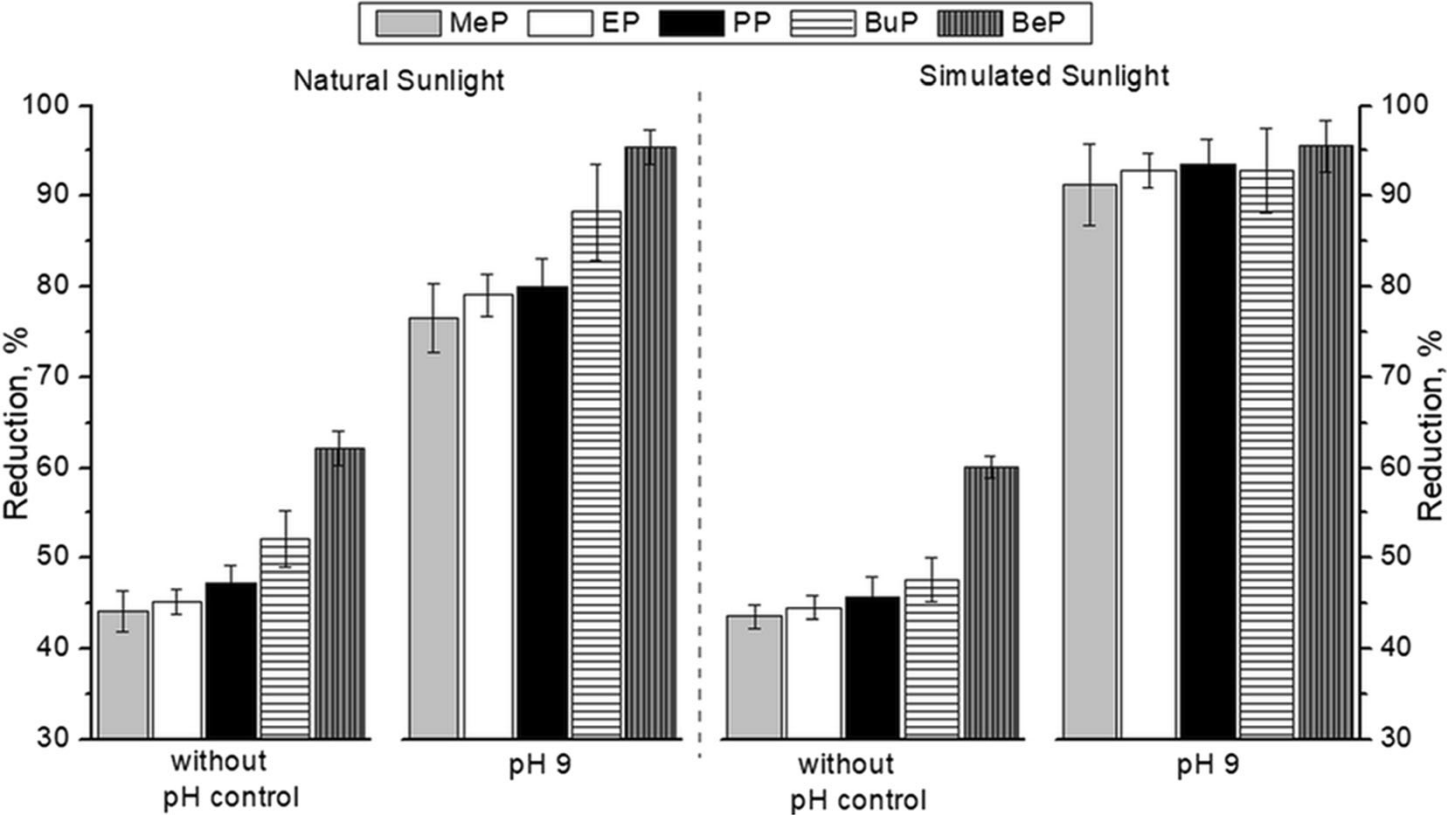
Degradation of parabens in MQ water during the sun and lamp irradiation in a buffered solution and without pH correction (after 180 min of reaction)

The efficiency of paraben mixture photodegradation by simulated (lamp) and natural sunlight radiation was investigated in various water matrixes. The experiments were carried out at pH 9 in buffer solution and in MQ water without pH control. pH 9 was selected based on our previous study (Gmurek et al. 2017), where it was found that under those conditions, the photodegradation occurred much faster. The same reaction solution was exposed to the simulated and solar light. The obtained results of paraben degradation after 180 min of photodegradation under various conditions are shown in Fig. 1.

In all tested solutions, after photosensitized oxidation process, partial degradation of all compounds was observed. As can be seen, the degradation of parabens is strongly correlated with their solubility (Table 1). The better solubility of the compound in water and the lower degree of its degradation were achieved. In all of the studied systems after 180 min of treatment, the MeP was the most resistant to degradation, but still at least 45% of this compound was degraded. The similarity of the chemical structure of the dissolved compound and solvent largely determines the solubility according to the principle of the *similia similibus solvuntur*. The lower affinity of the compound for water causes its higher adsorption on the surface of the catalyst support (Gmurek et al. 2017). It is known that both HO^·^ and ^1^O_2_ are short-lived individuals (Gmurek et al. 2017). Adsorption of a chemical compound on a carrier/catalyst that generates a reactive form of oxygen increases the likelihood of its oxidation. Observed difference between the efficiency of the photosensitization process in alkaline solutions and in solutions without pH control was about 30%. This effect may be probably due to the dissociation degree of the compounds (pK_a_). The values of pK_a_ of compounds used in the experiments are above 8, so in the solution without pH control, the compounds were predominant in undissociated form (Yalkowsky et al. 2010; Gmurek et al. 2015).

The type of light used during the photosensitizing oxidation process at the same pH solution does not significantly influence the efficiency of degradation. Degradation of the parabens in experiments with simulated solar radiation occurs evenly for all used compounds. Even so, for the parabens with lower molecular weight in buffered conditions, the degradation efficiency is somehow higher when artificial light was applied.

**Fig. 2:**
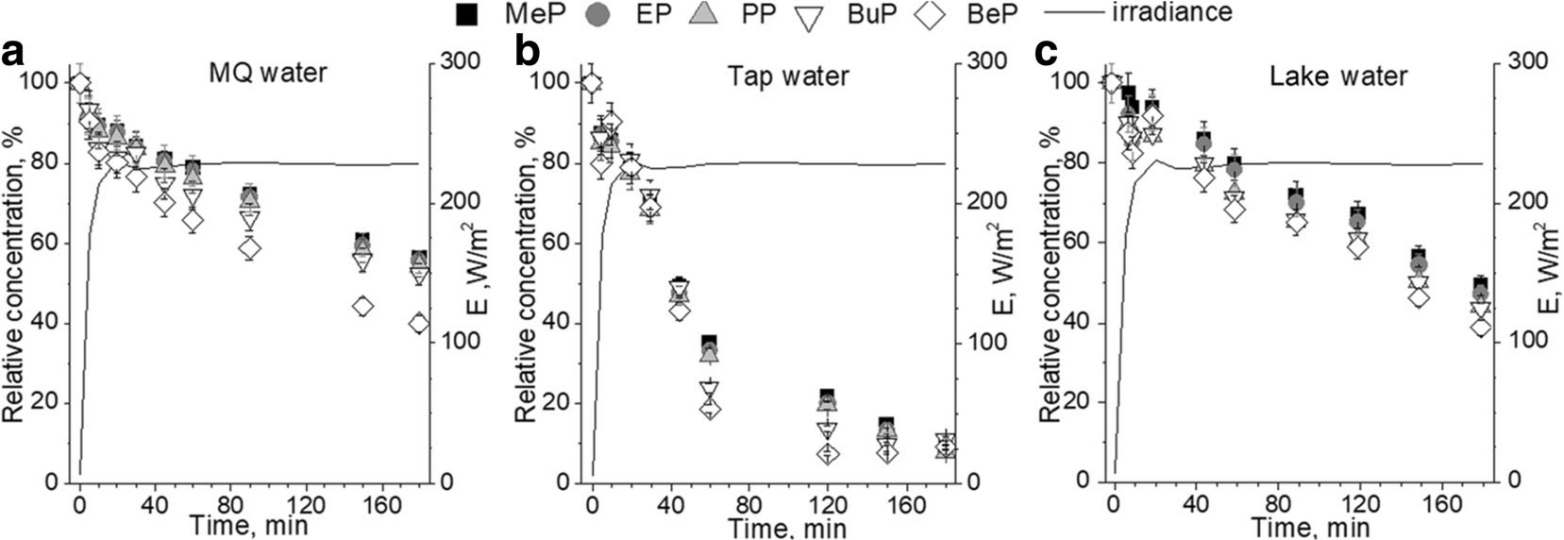
Photosensitized oxidation under simulated sunlight radiation in different water matrix

Comparison of paraben degradation in various types of water during lamp irradiation is presented in Fig. 2A–C. The average value of irradiance under simulated lamp radiation was 222.6 W/m^2^. The results achieved during the photosensitized oxidation process in MQ water (Fig. 2A) and lake water (Fig. 2C) showed no significant differences regarding the efficiency of paraben degradation. In MQ water as well as in lake water, BeP degradation was about 60%. Considering other tested parabens, the degradation in lake water was about 10% higher than for the same process in MQ water. The most effective process was the one where tap water was used as a matrix. For those conditions, the degradation of all five parabens was about 90%.

### Photocatalytic Oxidation

**Fig. 3:**
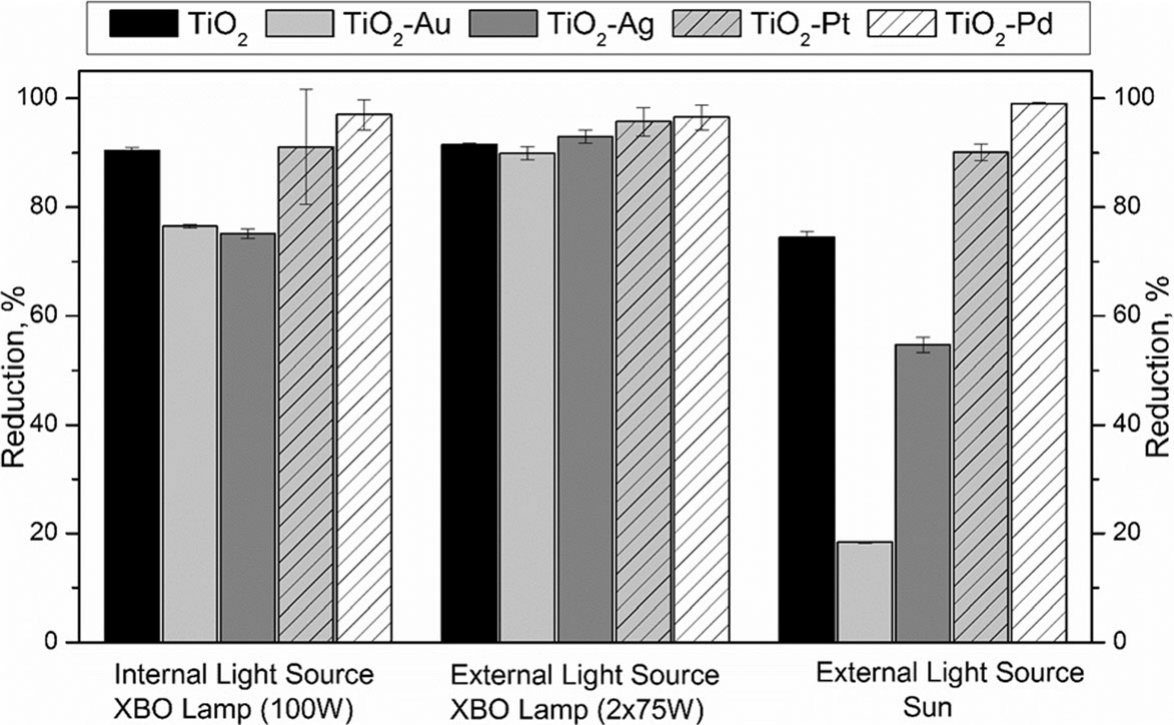
Photocatalytic degradation of parabens mixture under various light sources

The photodegradation process is influenced by the type of catalyst and intensity of radiation applied. Preliminary research has been carried out to check the applicability of TiO_2_ catalyst for the oxidation of parabens under sunlight. This part of the study was performed using solutions containing two parabens (MeP and EP) under simulated and natural sunlight. It is known that at low intensities of light, photocatalytic degradation rate increases linearly with increasing intensity (Petala et al. 2015; Doná et al. 2018). However, the influence of radiation intensity was never investigated in correlation with the type of reactor as well as the position of the light source. Figure 3 shows that the intensity of radiation influences the efficiency of photodegradation. The average degradation of parabens concentration was very similar for pure TiO_2_ and TiO_2_-Pd when internal as well as the external light source (Xe lamps) was considered. Meanwhile, for TiO_2_-Ag and TiO_2_-Au catalysts, better degradation was observed in the reactor with the external light source (2 × 75 W Xe lamps were used). When TiO_2_-Pt was applied, no influence on degradation was observed (in a reactor with internal and external light sources (Xe lamps) nor when as a light source sun was applied). It should be noticed that in the case of two reactors where Xe lamps were used, the light was entering the reactor via quartz so the UV light could reach the reaction solution. During natural sunlight experiments, glass reactor was used. The application of the glass reactor, which works as UV filter, allows to investigate only visible light effect on degradation. The efficiency of the photodegradation was much lower under natural sunlight, while under simulated sunlight, the degradation was very high.

**Fig. 4:**
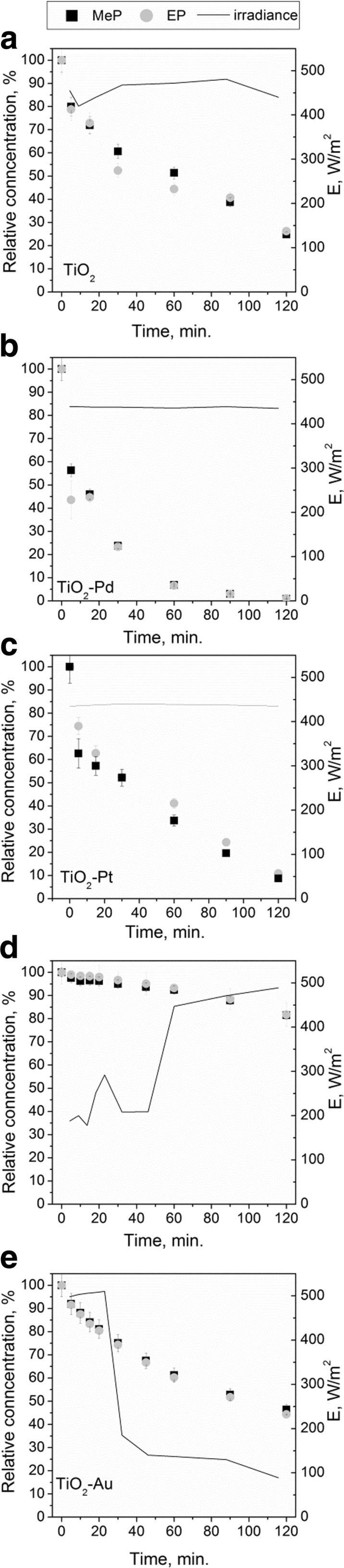
Photodegradation of parabens in MQ water under natural sunlight depending on the type of photocatalyst

To determine which of the doped TiO_2_ catalysts are the most efficient in the decomposition of 4-hydroxybenzoic acid derivatives during photodegradation process, additional studies have been conducted under natural sunlight. As can be seen in Figs. 3 and 4, the smallest percentage of degradation under sunlight was observed for TiO_2_-Au. Moreover, the doping with Ag was also ineffective in comparison to pure TiO_2_. A significant improvement was observed for TiO_2_-Pt and TiO_2_-Pd. It should be noticed that when TiO_2_-Pd was applied, the parabens were almost completely removed after 90 min. It should be noted that in Fig. 4D, E, the average value of irradiance was lower compared to cases A, B, and C, what in the case of Ag-doped TiO_2_ may have caused lower photodegradation efficiency of parabens mixture. However, the study indicates that catalyst doped by Au is not much photoactive in visible light and the high degradation under lamp irradiation was connected with UV light entering via the quartz wall of the reactor.

### Comparison of Both Processes

**Fig. 5:**
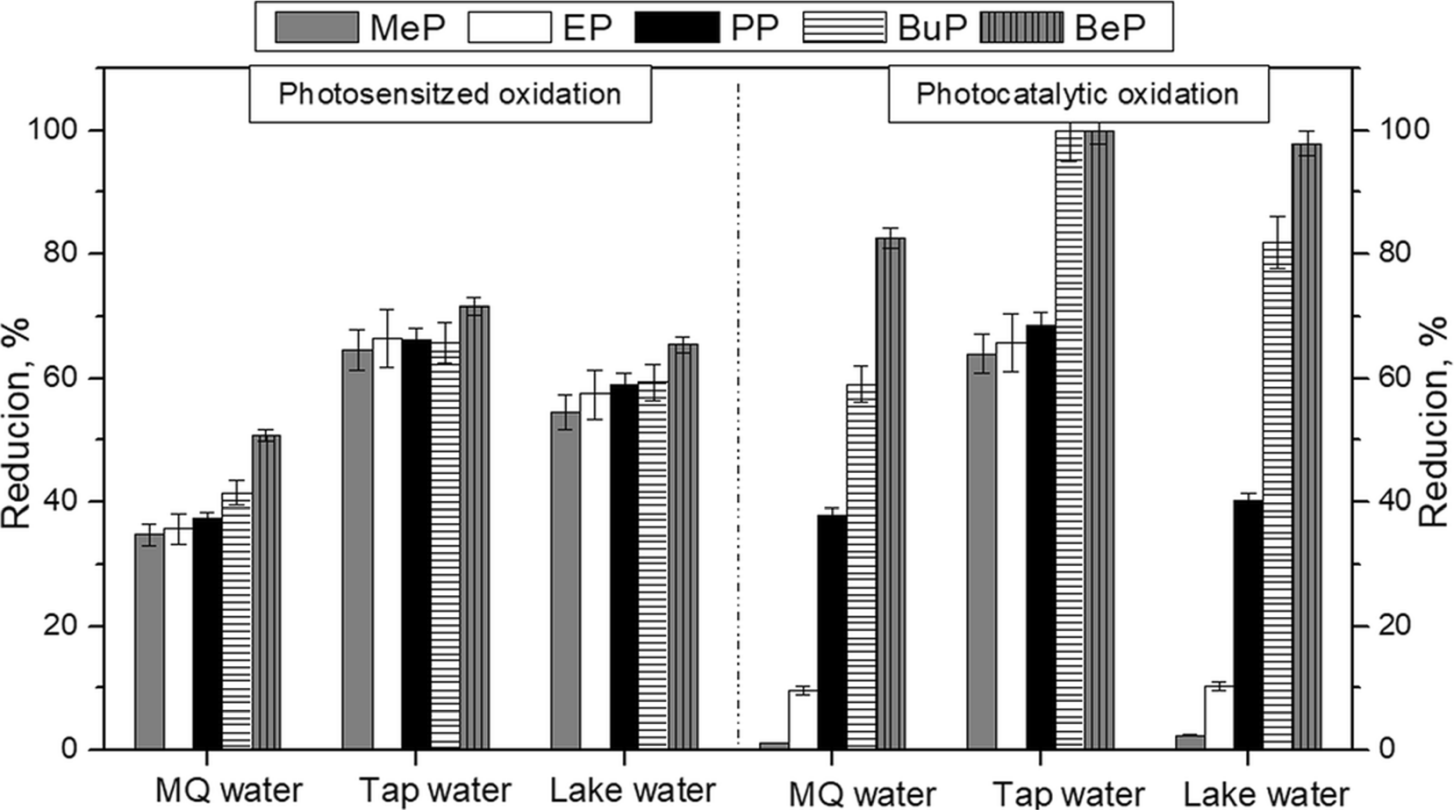
Comparison of photosensitized and photocatalytic degradation of parabens mixture in different water matrixes under natural sunlight

TiO_2_-Pd proved to be the most effective catalyst among the ones used for the degradation of parabens, which is why it was used in the subsequent studies. The photocatalytic oxidation experiments were carried out in the presence of 0.5 wt% TiO_2_-Pd. Studies on photocatalytic oxidation and photosensitized oxidation of parabens under sunlight were carried out simultaneously. During these studies, the effect of the solvent matrix on the efficiency of paraben degradation was also tested. The value of irradiance underwent a change in the course of the study, and its average value was 280 W/m^2^, 230 W/m^2^, and 340 W/m^2^ for experiment with MQ water, tap water, and lake water, respectively. The comparison of degradation efficiency for both photoprocesses is shown in Fig. 5. In both processes, the best degradation efficiency of parabens was obtained when tap water was applied as a matrix. The efficiency of paraben photodegradation increased with increasing length of the hydrocarbon chain, and the most effective degradation was for benzyl 4-hydroxybenzoate. As mentioned earlier, this could be caused by the highest adsorption of benzyl 4-hydroxybenzoate among the used parabens on chitosan/catalysts, what was presented in our earlier publication (Gmurek et al. 2017).

In order to try to understand the effect of the matrix on the processes efficiency, Table 2 presents the results of ion chromatography analysis performed for water matrixes.

**Table 2:** Ion chromatography analysis of water matrixes, mg/L

Ion	MQ water	Tap water	Lake water
F^−^	0.017	0.300	0.293
Cl^−^	0.079	10.961	14.331
Br^−^	0.075	0.082	0.085
NO_2_^−^	0.059	0.047	0.061
NO_3_^−^	0.121	3.357	0.109
PO_4_^3−^	0.146	0.200	0.247
SO_4_^2−^	0.075	27.536	38.562

The MQ water did not contain any other contaminants except for minimal content of some anions and cations. The TOC value for MQ water was ≤ 5 ppb. Tap water except the aforementioned anions may also contain a higher amount of iron cations and manganese compounds than MQ water (regulation restricts their content to 0.2 mgFe/L and 0.05 mgMn/L). Higher content of these ions can cause acceleration of degradation of parabens due to other ongoing processes along with photosensitized oxidation. Moreover, manganese compounds in the VII oxidation state exhibit oxidative properties, particularly strong in acidic environment. The lake water contained, besides the mentioned ions, humic acids, microorganisms, and other compounds that may affect the process. The more polluted matrix can affect the degradation efficiency of compounds during the conducted process in two ways. Firstly, it can accelerate the degradation process because other reactive oxygen species will appear in the solution. On the other hand, these pollutants can slow down the degradation of target compounds since they will scavenge or compete for the oxidants generated.

**Fig. 6:**
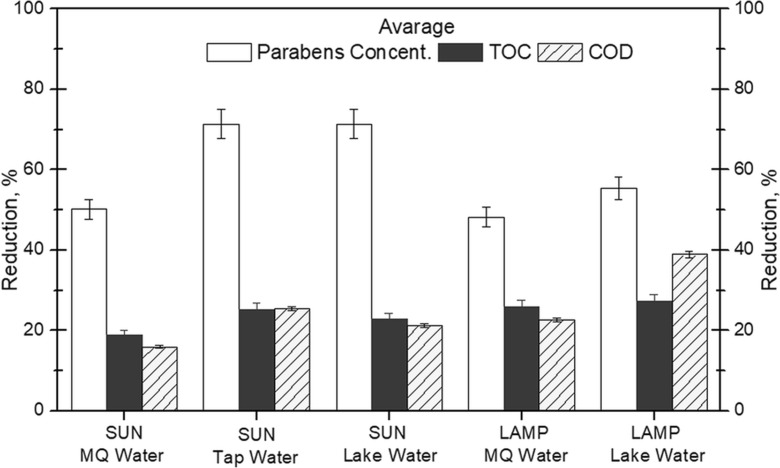
Reduction of TOC and COD values and an average reduction of parabens concentration after 180 min of photosensitized oxidation

The progress of the compounds mineralization was checked using TOC and COD analysis. TOC and COD results after photosensitized oxidation (Fig. 6) showed that the parabens were transformed into other chemical compounds and were not fully mineralized.

Although the highest average paraben concentration loss was observed in systems where sunlight was used, the obtained TOC and COD depletions did not exhibit the same efficiencies. In systems where solar radiation was used, reduction of TOC and COD was from 16 to 25%, while when lamp light was applied, the reduction was found to be from 23 to 39%. The highest TOC and COD reduction was achieved in the system using the lamp and lake water as matrix. The obtained degradation values were 27.5% and 39%, respectively. The worst reduction of these parameters was in the system involving sunlight and MQ water. This means that some compounds or ions contained in lake water promote the process of mineralization of parabens.

In case of photocatalytic oxidation, the higher TOC and COD reduction was achieved for experiments conducted in tap water. However, the TOC reduction was significant (34%), and the COD reduction was less than 10%. In lake and MQ water, the decrease of COD was similar, while TOC reduction was higher in lake water (18%) than in MQ (10%).

It should be noticed that lower degradation of parabens mixture was observed during 3 h of UVA photocatalytic oxidation (Gomes et al. 2017). The results proved that application of sunlight for photocatalytic oxidation of paraben mixture improved not only decontamination but also mineralization.

### Ecotoxicity Assessment

Ecotoxicity of the treated samples was investigated to estimate which process is better from an environmental point of view. The results of toxicity assessment are presented in Table 3.

**Table 3:** EC_50_ of reaction solution towards *V. fisheri* before and after photodegradation of five parabens mixture in the lake water under sunlight

Process	EC_50_ (%) before and after processes	
	Before	After
Photosensitized oxidation	0.3638 (0.2978–0.4443)	1.503 (1.397–1.616)
Photocatalytic oxidation	0.5058 (0.4030–0.6349)	8.645 (6.702–11.15)

Values in parenthesis are 95%CI

The toxicity decreased during both processes even though not complete removal of parabens was achieved. It is known that benzyl 4-hydroxybenzoate (BeP) is the most toxic paraben (Martins et al. 2016) and, as it was shown, the highest degree of degradation in both photoprocesses was achieved for it (Fig. 5). Therefore, it is expectable that when its degradation occurs, the toxicity of the mixture is reduced. During photosensitized oxidation, the decomposition of benzyl 4-hydroxybenzoate was about 65%, while during photocatalytical degradation, it was even higher.

### Microbiological Water Quality Analysis

**Fig. 7:**
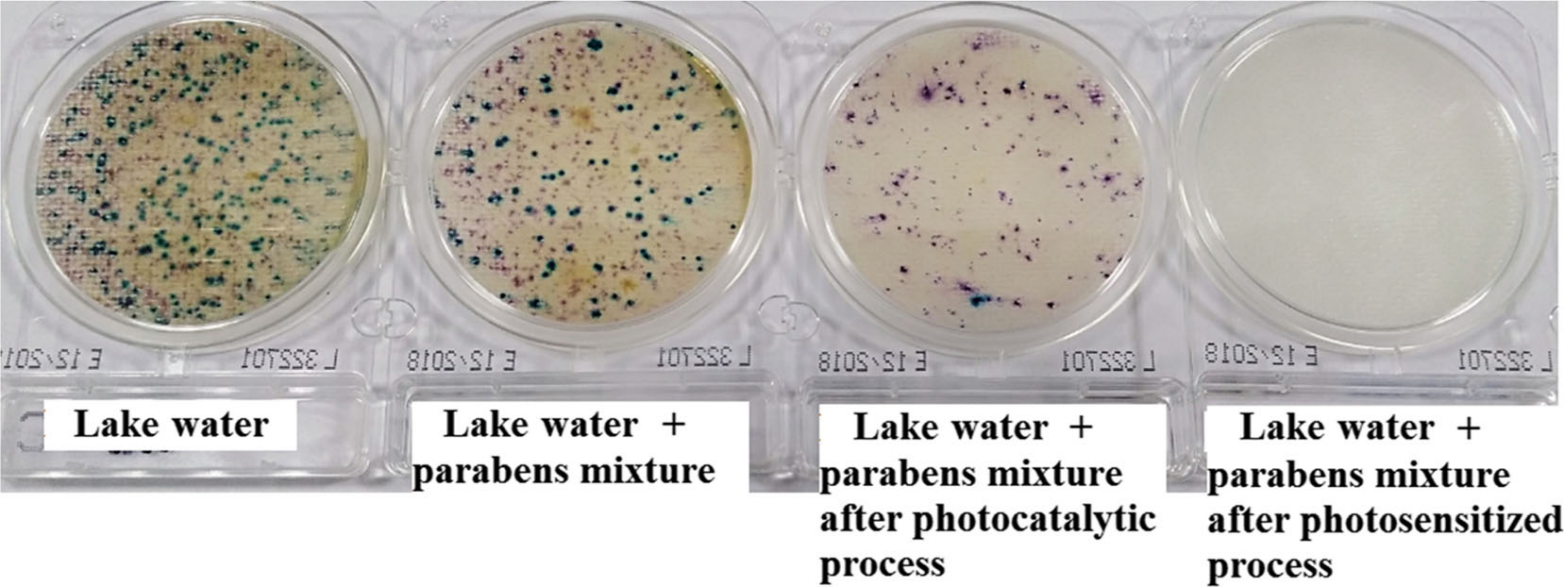
Results of compact dry tests for lake water and before and after processes

It is well known that the efficiency of water disinfection processes, especially solar disinfection, is monitored by measurements of bacteria using traditional plate count techniques (Berney et al. 2006; Boyle et al. 2008; Ghanizadeh et al. 2015; Castro-Alférez et al. 2016). Compact dry is a fast microbiological test ready for immediate use. Sterile scales include a substrate that allows selective growth of a particular microorganism group and their unambiguous identification. As can be seen in Fig. 7 in the lake water, there are many impurities—there are numerous colonies of coliform and small amounts of *Salmonella* bacteria on the plates. Moreover, the amount of *E. aerogenes* (red) is higher than *E. coli* (the blue one). Table 4 shows the results obtained for lake water with and without paraben mixture and after photocatalytic (TiO_2_-Pd) and photosensitized oxidation.

**Table 4:** Total bacterial cell concentration of lake water samples, water with paraben mixture, and after the reactions

The type of water	Compact dry CE CFU/ml			Compact dry SL CFU/ml
	*E. aerogenes* (red)	*E. coli* (blue)	Total coliform	*Salmonella*
Lake water	1400	130	1530	85
Lake water + parabens mixture	390	86	476	43
Lake water + parabens mixture after photocatalytic process (120 min)	99	32	131	24
Lake water + parabens mixture after photosensitized process (120 min)	0	0	0	0

The lake water (collected in July) with the naturally occurring coliform (1530 CFU/ml) and *Salmonella* (85 CFU/ml) was evaluated. Moreover, as expected, the addition of parabens to the lake water solution resulted in the three- and two-fold reduction of CFU/ml concentration for coliform and *Salmonella*, respectively. Furthermore, after the photocatalytic process, the number of colonies decreased by almost 12 times. The *Salmonella* seems to be more resistant and the total cell concentration of it dropped only twice. However, it should be noticed that after photosensitized oxidation, none of the bacteria was observed.

The use of compact dry as a microbiological test during water quality control showed effective destruction of coliform as well as *Salmonella* during solar oxidation (both photosensitized and photocatalytic). The singlet oxygen could be a better disinfectant because the membrane of the cell of microorganisms is the likely target for ^1^O_2_ oxidative reactions (Stief 2003; Malato et al. 2016). It could be concluded that photosensitized oxidation can be used for complete disinfection of water, as it imposes high oxidative stress on the cells and eventually compromises their vital functions, viability, and induces lethal damages.

## Conclusion

The studies show the possibility of photosensitized as well as photocatalytic oxidation of aqueous pollutants under natural as well as simulated sunlight. The effectiveness of the parabens degradation is affected by the intensity of radiation. Application of natural sunlight for both photochemical processes led to depletion of paraben concentration. However, it should be noticed that TiO_2_ modification by gold was not effective in the photodegradation of paraben mixture. The oxidants were identified as singlet oxygen and hydroxyl radicals for photosensitized and photocatalytic oxidation, respectively. The effectivity of both processes was comparable. The application of natural sunlight for the photosensitized oxidation of parabens led to water disinfection, while photocatalytic did not. Moreover, nanocatalyst separation from water suspension is not as easy as separation of chitosan beads of few millimeters in diameter. The photosensitized oxidation seems to be a promising and forward-looking water and wastewater purification method.

## Electronic Supplementary Material

ESM 1 (DOCX 178 kb)
