# Supplementary material for: Comparison of Photocatalytic and Photosensitized Oxidation of Paraben Aqueous Solutions Under Sunlight
Source: Water Air Soil Pollut. 2018 Oct 26;229(11):362. doi: 10.1007/s11270-018-3991-y (PMC6208757; doi:10.1007/s11270-018-3991-y)
Supplement: Supplementary file 1 — (DOCX 178 kb) [file 11270_2018_3991_MOESM1_ESM.docx]

COMPARISON OF PHOTOCATALYTIC AND PHOTOSENSITIZED OXIDATION OF PARABENS AQUEOUS SOLUTIONS UNDER SUNLIGHT

**M. Foszpańczyk^a#^, K. Bednarczyk^b^, R. C. Martins^c^, S. Ledakowicz^a^, M. Gmurek^a, c^**

^a^ Department of Bioprocess Engineering, Faculty of Process and Environmental Engineering, Lodz University of Technology, 90-924 Lodz, Wolczanska 213, Poland

^b^ Department of Safety Engineering, Faculty of Process and Environmental Engineering, Lodz University of Technology, 90-924 Lodz, Wolczanska 213, Poland

^c^ CIEPQPF – Chemical Engineering Processes and Forest Products Research Center, Department of Chemical Engineering, Faculty of Sciences and Technology, University of Coimbra, Rua Sílvio Lima, 3030-790 Coimbra, Portugal

**^#^**corresponding author: mfoszpanczyk@gmail.com

**
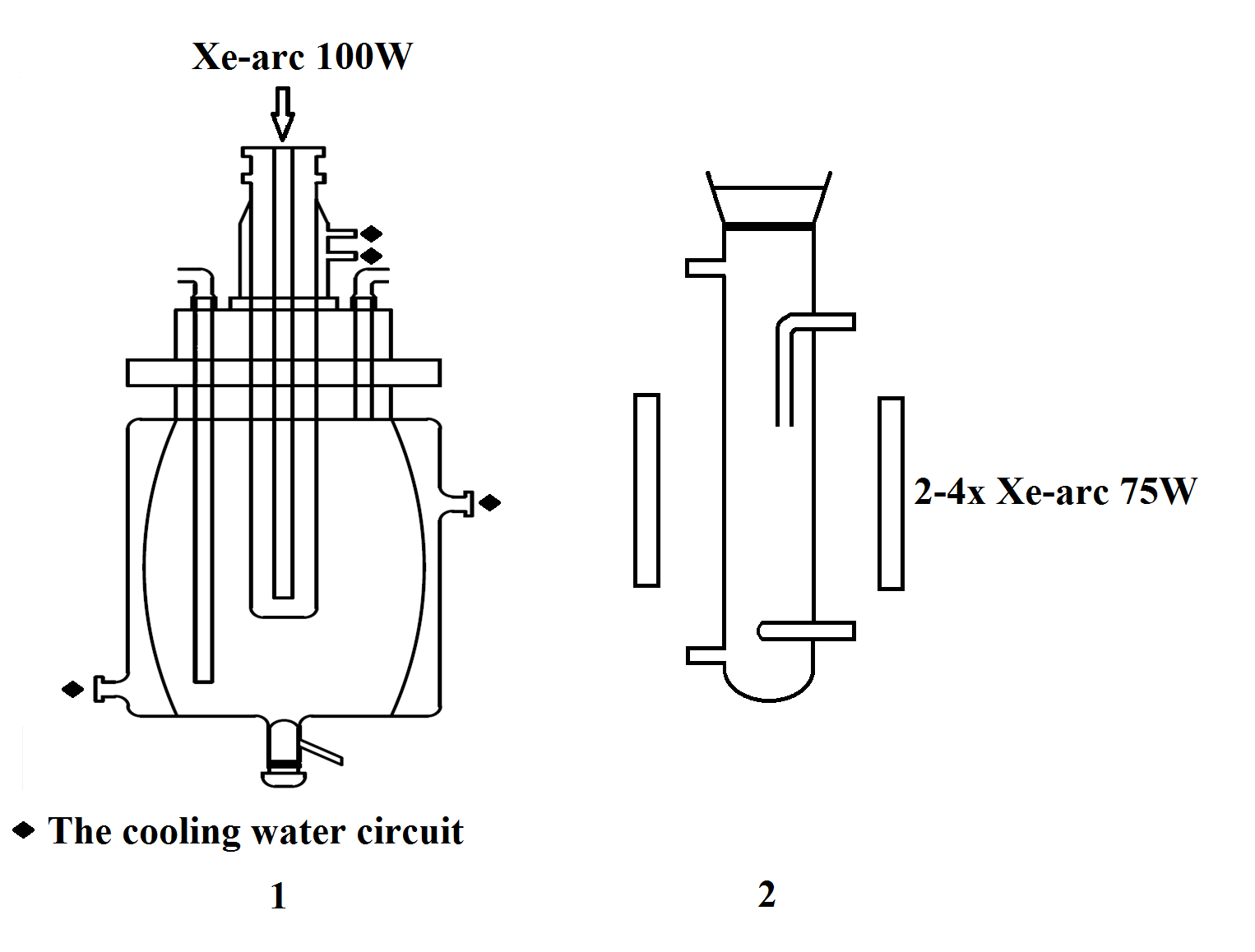

Fig. S1 The scheme reactor with cooling (1) and reactor with external radiation (2)**


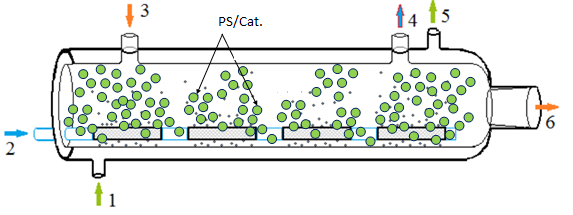
 **Fig. S2 The scheme of the 3^rd^ reactor applied for photosensitized experiments with lamp and for both processes sunlight experiments 1. cooling water inlet 2. gas supply pipe, 3. reactor filling location, 4. oxygen sampling and outlet, 5. cooling water outlet, 6. reactor drain.**

**Table S1. Analytical conditions used for quantification of selected parabens**

| Eluent A: 0.1 % Formic acid in methanol  Eluent B: 0.1 % Formic acid in water  Flow: 0.7 mL/min  Injection volume: 10 μL | | |
| --- | --- | --- |
| Time [min] | A [%] | B [%] |
| 0 | 30 | 70 |
| 1.5 | 15 | 85 |
| 2 | 30 | 70 |
| 3.5 | 60 | 40 |
| 6 | 60 | 40 |
| 12.5 | 100 | 0 |
